# La pubalgie du sportif de haut niveau: place du traitement chirurgical, à propos d'une série continue de 100 cas

**DOI:** 10.11604/pamj.2014.19.4.3294

**Published:** 2014-09-02

**Authors:** Jalal Boukhris, Rifi Mojib, Sami Mezghani, Jean Henri Jaeger

**Affiliations:** 1 Service de Chirurgie Orthopédique du Genou et Traumatologie du Sport, CHU Strasbourg, France

**Keywords:** Pubalgie, sportif, chirurgie, Pubalgia, sports, surgery

## Abstract

Parmi tous les syndromes de surmenage liés à la pratique sportive, la pubalgie est certainement un des plus invalidants. C'est un syndrome douloureux de la région inguino-pubienne qui touche particulièrement le joueur du football. Les formes inguino-pariètales sont de loin les plus fréquentes. Le traitement médical est entrepris dans la majorité des cas, mais il semble être moins efficace dans ce sport en particulier. La chirurgie occupe une place prédominante dans la prise en charge. Nous rapportons l'expérience de la prise en charge chirurgicale de 100 sportifs de haut niveau au sein de notre établissement, selon la technique de Nesovic, ainsi que leurs devenirs sportifs et nous discutons nos résultats en se comparant aux données de la littérature récente.

## Introduction

La pubalgie est un syndrome douloureux de la région inguino-pubienne qui touche le sportif de pratique régulière notamment le joueur du football. Il s'agit d'une pathologie de surmenage survenant dans un contexte anatomique et biomécanique particulier. Les formes inguino-pariétales sont de loin les plus fréquentes. La chirurgie occupe une place prédominante dans la prise en charge. Nous rapportons notre expérience de la prise en charge chirurgicale de 100 sportifs de haut niveau, selon la technique de Nesovic ainsi que leurs devenirs sportifs.

## Méthodes

**Description de la technique [1]:** selon Nesovic, les pubalgies étant secondaires à un déséquilibre entre une musculature abdominale déficiente et souvent hypotonique et des adducteurs de la hanche plus « forts », il a eu recours à une intervention de rééquilibrage par plastie abdominale qui cherche à pallier l'insuffisance des muscles obliques et à obturer sous tension la déhiscence, véritable hernie directe, située entre le bord inférieur du tendon conjoint et le ligament inguinal. Cette intervention permet de stabiliser la symphyse pubienne en s'opposant à l'action prédominante des adducteurs. Elle se rapproche de la cure de hernie inguinale selon Bassini. L'intervention comporte deux temps opératoires.

**Figure 1:**
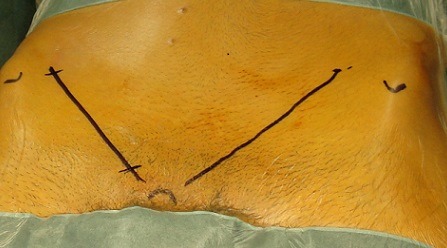
Tracé d'incision pour intervention de Nesovic

**Figure 2:**
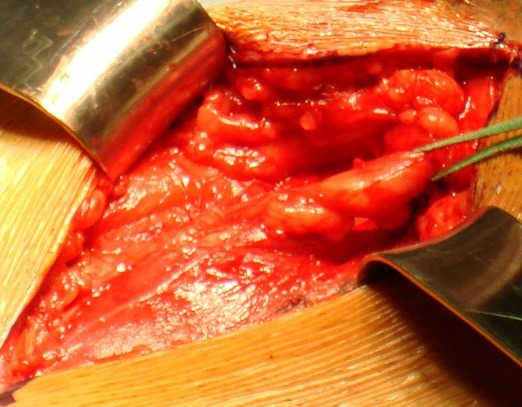
Dissection du cordon spermatique

**Le temps de dissection**: le patient est installé en décubitus dorsal. L'incision débute au niveau de l’épine du pubis et se dirige vers l’épine iliaque antéro-supérieure, pour se terminer à deux travers de doigt en dedans d'elle (Figure 1). Après hémostase soigneuse du tissu sous-cutané, on décolle au tamponmonté la face superficielle de l'aponévrose de l'oblique externe qui, bien souvent, est le siège de fissurations longitudinales d’étendue variable. L'incision de l'aponévrose de l'oblique externe est réalisée dans le sens des fibres, en général dans une zone de fissuration aponévrotique si elle existe, depuis l'orifice superficiel du canal inguinal jusqu'au niveau de l’épine iliaque antéro-supérieure. Les deux faces profondes de l'aponévrose incisée sont alors décollées de haut en bas jusqu’à l'arcade crurale. Le cordon inguinal est alors repéré, disséqué et mis sur un lac (Figure 2). Après cette dissection, on constate constamment l'existence d'une déhiscence entre le bord inférieur du tendon conjoint et l'arcade crurale.

**Le temps de réparation**: ce temps comporte une réparation profonde appelée par Nesovic myofascioplastie, suivie d'une réparation superficielle qu'il a dénommée fascioplastie.

**Figure 3:**
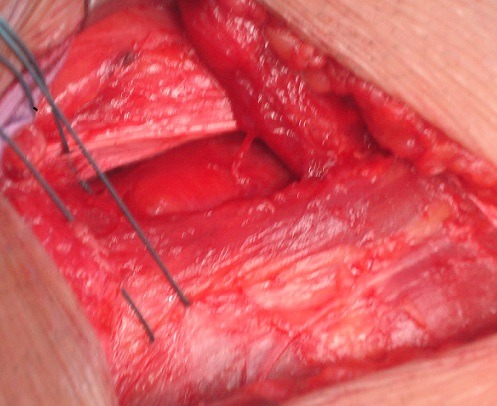
Myofascioplastie profonde (a)

**Figure 4:**
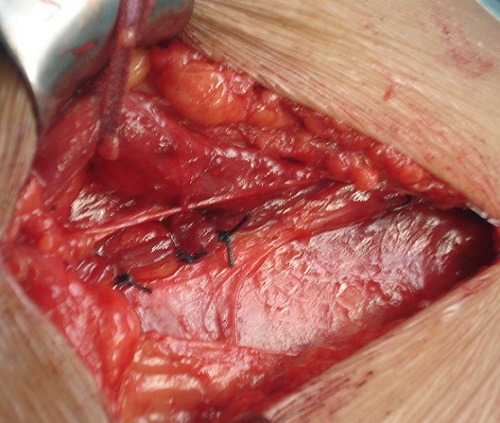
Myofascioplastie profonde (b)

*Myofascioplastie profonde:* l'extrémité inférieure du grand droit ainsi que l'extrémité toute inférieure du tendon conjoint sont chargées par un fil non résorbable qui sera amarrée au niveau du périoste du pubis (Figure 3, Figure 4). Ainsi, de proche en proche, par serrage des fils, la paroi abdominale est amenée au contact de l'arcade crurale.

**Figure 5:**
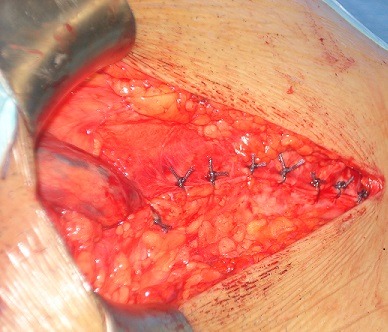
Fascioplastie superficielle

*Fascioplastie superficielle:* l'aponévrose de l'oblique externe est suturée sans excès de tension. Le cordon inguinal peut être maintenu en position pré-aponévrotique (Figure 5). Nous attirons l'attention sur deux pièges à éviter: le serrage trop important des sutures pouvant être source de douleurs traînantes; la compression du cordon avec le risque extrême de nécrose testiculaire. La phase de récupération postopératoire consiste en quatre semaines de repos. Au terme de ce délai, une remusculation isométrique des muscles larges de l'abdomen, la pratique du vélo, la natation, le footing peuvent être autorisés. La reprise de l'entraînement au sport de compétition ne sera autorisée que si les tests d'adduction contrariée se sont négativés, soit en moyenne vers la sixième semaine postopératoire pour les entraînements et la dixième semaine pour la compétition.

**Description de la série**: il s'agit d'une étude rétrospective portant sur 100 patients opérés au sein de notre établissement selon la technique de Nesovic entre Janvier 2005 et Décembre 2011. Notre série se composait exclusivement d'hommes, et majoritairement de footballeurs de compétition. Aucun patient n'a été opéré avant un délai de trois mois à partir des premiers signes, ce qui correspond à une épreuve de traitement médical dont la base est le repos sportif. Les détails techniques opératoires ont été soigneusement notés, ainsi que les traitements complémentaires et la chronologie de la reprise des activités sportives.

## Résultats

Le sport pratiqué par les sportifs de notre série est principalement le football (80%) et se sont dans 89% des joueurs professionnels ou de haut niveau. L’âge moyen lors de l'intervention chirurgicale était de 24 ans (14 à 37 ans). Les formes bilatérales représentaient 70%. L'association d'une atteinte des adducteurs a été décelée dans 60% des cas. L'intervention a été réalisée bilatéralement dans 85% dans cas. Une ténotomie du long adducteur s'est avérée nécessaire chez uniquement 10% des patients. En postopératoire, nous avons déploré cinq hématomes dont deux repris chirurgicalement et ayant évolué favorablement. Avec un recul moyen dépassant les cinq ans (29 à 97mois) les résultats sur les douleurs ont montré une disparition complète des symptômes chez 76% des patients. La reprise des entraînements a été effectuée vers la 18^ème^ semaine en moyenne. La reprise de la compétition a nécessité quatre semaines de plus. Le taux de reprise du football au niveau antérieur avoisinait les 90%.

## Discussion

L'ensemble de la littérature s'accorde à penser que le traitement médical est le traitement de première intention d'une pubalgie. Le traitement conservateur permet une guérison dans 80 à 85% des cas [1]. Dans les formes rebelles à un traitement bien conduit pendant trois mois en moyenne, une prise en charge chirurgicale peut être proposée. Le traitement chirurgical s'adresse particulièrement au footballeur de haut niveau après 3 mois (mais pas après 8 mois) de traitement médical bien conduit et surtout s'il possède une paroi abdominale tonique [2], un orifice inguinal superficiel distendu et très douloureux. La technique retenue est celle de Nésovic, souvent pratiquée par des chirurgiens orthopédistes et qui garde l'avantage de sa simplicité, des suites plus confortables avec des délais de reprise plus raisonnables [1]. Les techniques endoscopiques d'obturation simple par des renforts prothétiques, pratiquées par des chirurgiens viscéraux, théoriquement moins agressifs, n'ont pas encore réussi à faire la différence; ces prothèses sont complètement déconseillées par la quasi-totalité des chirurgiens du sport. Ces techniques ne répondent pas au souci de rééquilibrage de la symphyse pubienne.

Le maintien d'un traitement conservateur pendant 3 mois avant de poser l'indication dans les formes touchant le canal inguinal est partagé par d'autres auteurs [3–8]. Wodecki [8] précise que ce traitement s'adresse aux formes pariéto-abdominales pures ou prédominantes. Gilmore [9] lui, sur plus de 1000 cas depuis 1980 préconise un traitement chirurgical des souffrances du canal inguinal après échec d'un programme de réadaptation de 4 semaines. Pour Baquie [10], en revanche, la chirurgie est utilisée dans un très petit nombre de cas à la fin de la seconde saison douloureuse seulement.

Enfin, plusieurs auteurs posent l'indication d'emblée lorsque le diagnostic de forme inguinale de pubalgie est posé. Vidalin [11] nous rapporte 72 cas. Il pose une indication chirurgicale sans réserve lorsqu'il existe une pathologie clinique de l'orifice herniaire avec douleur au toucher inguinal. L'indication est portée, en revanche, avec réserves lors de pathologie rachidienne associée, d'arthropathie pubienne ou de pathologie des adducteurs. Mais l'indication chirurgicale lui semble d'emblée la meilleure s'il existe une lésion pariéto-abdominale. De même, Ekstrand [12], en 2001, montre dans sa série prospective randomisée de 66 cas la supériorité du traitement chirurgical d'emblée ou différé dans les pubalgies rebelles liées à une souffrance du canal inguinal.

Biedert [13] dans une étude de plus faible importance portant sur 24 athlètes évalués jusqu’à 6,6 ans après le geste chirurgical effectué en moyenne 17 mois après le début de la symptomatologie a lui aussi des résultats excellents avec des scores subjectifs de 10,2 sur une échelle de 12; et des scores objectifs de 12 sur un maximum de 13. La reprise de toutes les activités sportives a été possible chez 23 des 24 athlètes opérés. Plus ou moins souvent associé à un traitement pariétal, un geste de l'autre côté du carrefour pubien, en l'occurrence la ténotomie des adducteurs, est à reléguer au second plan, car considérée comme trop délabrante pour les sportifs de haut niveau et pouvant être source d'hypotonie musculaire [14].

Akermark a obtenu uniquement 62,5% de bons résultats sur 16 ténotomies [15]. Nous pensons qu'il s'agit dans la majorité des cas d'un phénomène purement fonctionnel qui ne requière pas d'indication opératoire. Elles doivent être réservées aux formes à symptomatologie prédominante au niveau des adducteurs, aux calcifications ou séquelles de claquages, ou bien en cas de douleurs persistantes des adducteurs après une plastie abdominale de type Nesovic.

La rééducation postchirurgicale doit respecter les délais de cicatrisation de la sangle abdominale, ce qui demande d’éviter toutes sollicitations intempestives de la paroi pendant trois à quatre semaines. Puis on réalisera un travail d'assouplissement des cicatrices (massages et ultrasons), une tonification douce de la sangle abdominale qui peut débuter par une électrostimulation et des contractions isométriques et des étirements et un renforcement des adducteurs. Puis on réalise une reprogrammation neuromusculaire avant de réintroduire le footing aux alentours de la sixième semaine postopératoire et le travail des gestes techniques spécifiques vers la huitième semaine.

## Conclusion

La pubalgie du footballeur est actuellement une entité bien connue, dont prise en charge ne fait pas encore l'objet d'unanimité. La chirurgie occupe une place prédominante dans le traitement. Les techniques chirurgicales sont nombreuses dans le traitement de l'insuffisance pariétale. La technique de Nesovic mérite une place de choix dans la mesure où les résultats sont constamment excellents, lorsque les indications sont bien posées.
